# Changes in sugar-sweetened beverage purchases across the price distribution after the implementation of a tax in Mexico: a before-and-after analysis

**DOI:** 10.1186/s12889-023-15041-y

**Published:** 2023-02-07

**Authors:** J. C. Salgado Hernández, S. W. Ng, M. A. Colchero

**Affiliations:** 1 National Council for Science and Technology and Center for Nutrition and Health Research, Instituto Nacional de Salud Pública, Universidad No 655 Colonia Santa María Ahuacatitlán, Cerrada Los Pinos Y Caminera. CP 62100, Cuernavaca, Morelos México; 2 Department of Nutrition, Gillings School of Global Public Health, University of North Carolina at Chapel Hill and Carolina Population Center, CB #8120, 123 West Franklin Street, Chapel Hill, NC 27516 USA; 3 Center for Health Systems Research, Instituto Nacional de Salud Pública, Universidad No 655 Colonia Santa María Ahuacatitlán, Cerrada Los Pinos Y Caminera. CP 62100, Cuernavaca, Morelos, México

**Keywords:** Sugar-sweetened beverages, Taxes, Purchases, Prices, Mexico

## Abstract

**Background:**

A tax of one-Mexican peso per liter of sugar-sweetened beverage (SSB) came into effect in January 2014 in Mexico as a national health policy to tackle the high overweight and obesity prevalence. Previous studies have shown an overall reduction in SSB purchases after the tax implementation. However, it remains unknown whether and to what extent SSB consumers switched to cheaper taxed beverages, attenuating the potential effect of the policy. Our study’s objective was to estimate changes in household purchases of taxed SSBs by tertiles of SSB prices (low, middle, and high) in urban areas after the SSB tax implementation in 2014.

**Methods:**

Based on purchase data for 2012–2015 from households living in 54 Mexican cities with a population > 50,000 inhabitants, we calculated unit-value SSB prices for the full period and sorted them on a monthly basis to create monthly price tertiles. We merged these price tertiles to household purchases and created average monthly ml/capita/day SSB purchases by price tertile at the city level. We assessed SSB purchase switching patterns before and after the tax implementation through price-tertile stratified linear models. The main variable in the models was a dummy indicator that allowed us to identify the pre-tax period (2012–13) and post-tax period (2014–15). We controlled our models for time trends and contextual economic variables.

**Results:**

In the regression adjusted models, we found a statistically significant purchase reduction ranging between 10.80 and 13.79 ml/capita/day (*p*-value < 0.001) across taxed beverages from the middle-price SSB after the tax implementation. We observed no statistically significant reductions in purchases of low-price SSBs and high-price SSBs.

**Conclusions:**

Our findings show purchase reductions in the middle-price SSBs, which represents ≈30% of the overall SSB purchases in urban Mexico. Future studies should be conducted to test if the redesign of the current the tax, by either doubling the tax amount or taxing sugar content, might reduce more effectively purchases across all SSBs.

**Supplementary Information:**

The online version contains supplementary material available at 10.1186/s12889-023-15041-y.

## Background

In 2014, Mexico implemented a tax of one Mexican peso per liter on sugar-sweetened beverages (SSB) as a public health policy to tackle the high prevalence of overweight and obesity, which reached 35% for children and teenagers and 71% for adults [1]. This SSB tax represented about a 10% average increase in SSB prices. One year after the tax implementation, evaluation studies showed reductions in taxed beverage purchases by 4% in rural areas and 6.3% in both urban areas and at the country level [2, 3]. In urban settings, the reduction in taxed beverage purchases ranged between 8–17% two years after the SSB tax implementation [4, 5]. However, it remains unknown whether and to what extent SSB consumers switched to cheaper taxed beverages, attenuating the potential effect of the policy.

Differential reductions in SSB purchases would be expected in Mexico, given the large SSB price variation ranging from four to sixteen Mexican pesos (MP) per liter [6]. Given this price-related dispersion, consumers could have switched to cheaper SSB options as a response to the SSB tax to minimize their reduction in SSB purchases. Evidence of heterogeneous purchase reductions across the SSB price distribution might indicate that the current one-Mexican peso tax is too low to reduce the price gap across the SSB price distribution. Thus, the recommended tax by public health experts of two Mexican pesos [7] might be better suited for shrinking the price gap across SSBs by imposing a relatively larger tax burden on larger package size SSB, which tend to be cheaper per liter.

Evidence from other countries shows differential reductions in purchases associated with taxes on SSBs by brands and package sizes and thus in prices. In a simulation-based analysis for the United States, Liu, Lopez, and Zhu [8] predicted that a tax of one cent per ounce of soft drink led to average reductions by 5.8% across regular soft drinks and 7% across diet soft drinks. However, there were heterogenous purchase reductions across producers and within their product portfolios. In the context of the 10% ad valorem SSB tax in Barbados, which came in effect in 2015, Alvarado et al. [9] analyzed the SSB sales reductions overall and by price tertile. The authors found an overall SSB sales reduction by 4.3% after the tax implementation. However, this reduction was 2.6 and 14.4% for low- and high-cost SSBs, respectively. In contrast, there was a sales increase of 4.3% among middle-cost SSBs. Likewise, Alvarado et al. [9] found sales increases by 6.5% for middle-cost carbonated SSBs and 17.6% for low-cost non-carbonated SSBs. For the remaining price tertiles within carbonated SSBs or low-cost non-carbonated SSBs, the authors found no change or sales reductions.

In Mexico, despite the sustained overall reduction of SSB purchases [4], the magnitude of this decrease across the SSB price distribution is unknown. The objective of the study was to estimate changes in household purchases of taxed beverages by tertiles of SSB prices (low, middle, and high) before and after the implementation of the tax, using data from Nielsen Mexico Consumer Panel Service, representative of urban areas, between 2012 and 2015.

## Methods

In this study, we defined SSBs as taxed beverages including industrialized flavored water, ready-to-drink tea, industrialized juices and nectars (excluding 100% juices), ready-to-drink sports drinks, and soft drinks. To estimate changes in SSB purchases by price tertile, we used several sources of data.

### Data

We retrieved information on SSB household purchases and SSB prices in 2012–2015 from the Nielsen Mexico Consumer Panel Service (Nielsen CPS) [10]. Nielsen CPS [10] is a panel of around 6,000 households across 54 cities and is representative of urban settings with a population larger than 50,000 inhabitants. This population accounts for 63% of the overall Mexican population and amounts to 75% of food and beverage expenditure in 2014 [11, 12]. Price and purchase information in Nielsen CPS comes from diaries, scanning of bar codes of product packaging from selected products (e.g., packaged food and beverages), receipts, and pantry surveys. This information is collected every two weeks by interviewers who attended sampled households [2, 10].

In addition to Nielsen CPS, we gathered contextual information from the National Institute of Statistics and Geography. Specifically, we used quarterly unemployment rate [13] and average monthly per capita labor income [14]. When possible, we merged this information at the city level to Nielsen CPS; otherwise, the merging procedure is at the state level where cities are nested in. We calculated real prices and income using the consumer price index in Mexico City in January 2012 as the reference [15]. Finally, we retrieved yearly population size at the city level from the National Population Council in Mexico [16], as weights for the models, as explained below.

### Empirical model

We assessed SSB purchase switching patterns before and after the implementation of the tax through price-tertile stratified linear regression models. We regressed monthly SSB purchases in milliliters (ml) per capita per day on a post-tax dummy variable, which equals one for periods in 2014–15 and zero otherwise, stratified by SSB price tertile.

To estimate SSB price tertiles, we adapted the methods used by Alvarado et al. [9] in the evaluation of the Barbados SSB tax. We first calculated a single real unit value (prices per liter) for each combination of SSB brand and package size for the full period of 2012–2015 in urban Mexico (all cities in Nielsen CPS). Unit values were estimated by dividing expenditures over quantity purchased for each combination. In Nielsen CPS [10], there were 1,421 unique combinations of brand and package size. Additional figure A1, shows how many months products are purchased in the analytical period (i.e., 48 months spanning from January 2014 to December 2019). This graph shows that urban households do not purchase all products across all months. Hence, for each month, we sorted the unit-value prices, calculated price tertiles, and merged them to households’ SSB purchases. By calculating price tertiles on a monthly basis, we account for the fact that households purchase some specific products for specific months.

Then, we calculated average ml/capita/day SSB purchases by price tertile (low, middle, and high) at the city level. Therefore, household data set was aggregated at the city/month/price tertile level. For the calculation of ml/capita/day purchases, we kept the representativeness of the data by using sampling weights among all households in the data regardless of the fact they reported or did not report purchasing products in the relevant price tertile. Our analytical data is composed of 7,776 observations resulting from one monthly observation (for each of the 48 months) at the tertile price level for each of the 54 cities in Nielsen CPS [10].

We adjusted the model for a set of control variables that may be associated with household purchases: quarterly season fixed effects, city fixed effects, a linear time trend (a count variable for month/year) along with its squared transformation, and contextual economic variables, i.e., per capita monthly labor income and unemployment rate. We weighted the linear regression models with the respective city’s population size and estimated robust standard errors. In our study, the main coefficient of interest corresponds to the post-tax period dummy variable that captures changes in ml/capita/day SSB purchases by price tertile before and after the SSB tax implementation. In an additional analysis, we controlled for the percentage of products within each price tertile of interest with a package size larger than one liter, which represents large package sizes that tend to exhibit lower prices per liter compared to smaller presentations. Thus, we are able to account for the relatively cheaper prices for large package sizes, which might drive their demand upwards.

We aggregated the data at the city/month/price tertile level instead of leaving it at the household level because, based on Handbury and Weinstein [17] who describe potential biases linked to price information at the household level. These biases correspond to searching behaviors by households looking to pay lower prices or retailers charging different prices for the same product. However, we provide a description of the distribution of price tertile by SES before and after the implementation of the tax using the household data.

To help interpret findings from the models presented above, we calculated pre- and post-tax prices. These pre- and post-tax prices provide evidence of the differential price increases by price tertile after the SSB tax implementation, that can be used to understand how these price increases drove purchase changes. We restricted these price calculations to products with available information for both pre- and post-tax periods. For these products, we calculated average prices for the full pre-tax period (i.e., 2012–2013) and sorted these prices to define the price tertiles for the complete period (2012–2015). Then, for each tertile, we calculated the average post-tax prices.

## Results

**Fig. 1:**
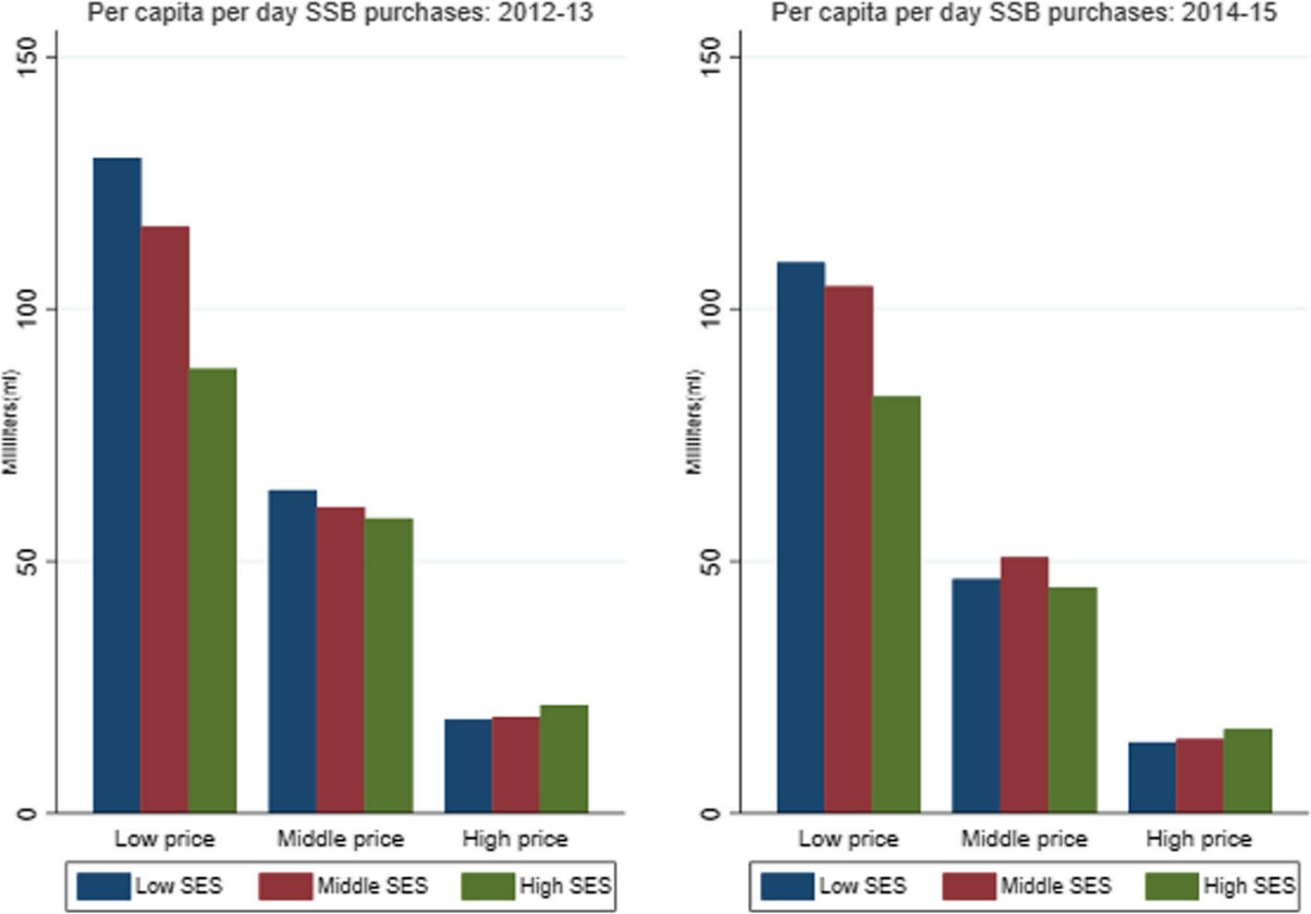
Sugar-sweetened beverage purchases by household socioeconomic status and price tertile. *Note:* SSB: sugar-sweetened beverage, SES: socio-economic status. Population weighted averages. Source: Authors’ own analyses and calculations based on data from Nielsen through its Mexico Consumer Panel Service (CPS) for the food and beverage categories for January 2012 –December 2015. The Nielsen Company, 2016. The conclusions drawn from the Nielsen data are those of UNC and do not reflect the views of Nielsen. Nielsen is not responsible for and had no role in, and was not involved in, analyzing and preparing the results reported herein

Figure 1 shows the average ml/capita/day SSB purchases by price tertile and households’ socioeconomic status (SES) (low, middle, and high). Regardless of SES, households purchased a larger volume of cheaper SSBs. However, in the pre-tax period, low-price SSBs are purchased more by low- and middle-SES households compared to high-SES households that tended to purchase a larger volume of high-price SSBs. The difference in purchases of middle-price SSBs is small across SES households. In the post-tax period, we observed similar trends as in the pre-tax period, with slight variations in the middle-price SSB. After the tax implementation, there was a drop in SSB purchases across all households for all socio-economic groups. Additional Table A1 shows that this drop in SSB purchases was statistically significant at the 1% level.

Table 1 shows summary statistics comparing all variables included in the model in the pre-tax period (2012–13) and the post-tax period (2014–15). As noted above, a negative relationship holds between price tertiles and SSB purchases, i.e., for low-price SSBs, beverage purchases are higher. Thus, the highest and lowest average SSB purchases correspond to the low- and high-price tertile, respectively. Across all SSB price tertiles, we see a SSB purchase decline after the tax implementation, but the highest percentage decline happened in the high-price SSB group (23%). Meanwhile, larger absolute declines (around 12 ml/capita/day) took place across low- and middle-price SSBs. This purchase decline between pre- and post-tax periods across SSB price tertiles is statistically significant at 1%. For the contextual variables at the bottom of Table 1, there was a decrease in real per capita income and unemployment rate by comparing the post-tax period to the pre-tax period. Across all price tertiles, soft drinks and industrialized juices and nectars concentrate between 85–99% of SSB purchases (results not shown). However, while soft drink purchases have a lower contribution as we move across price tertiles (92% for the low-price tertile and 56% for the high-price tertile), the opposite pattern holds for juices and nectars (7% for the low-price tertile and 30% for the high-price tertile). These contributions are similar over time.

**Table 1:** Summary statistics of the analytical sample at the city-month-price-tertile level

		**2012–13 (pre-tax)**		**2014–15 (post-tax)**		**2014–15 vs 2013–2012**	
		**Mean**	**SD**	**Mean**	**SD**	**Mean**	***P*-value**
**SSB purchases (ml/per capita/day)**	**Low- price SSB**	111.27	43.50	98.90	46.58	-12.37	0.00
	**Middle- price SSB**	59.87	46.47	48.02	26.23	-11.84	0.00
	**High- price SSB**	19.25	7.18	14.91	6.90	-4.33	0.00
**Contextual variables**	**Per capita labor income (MP$)**	2298.09	270.15	2172.93	291.85	-125.16	0.00
	**Unemployment rate (%)**	5.49	1.18	5.08	1.23	-0.41	0.00
	**Observations**					7776	

Population weighted summary statistics. Standard deviations in parentheses. MP: Mexican pesos. *Source:* Authors’ own analyses and calculations based on data from Nielsen through its Mexico Consumer Panel Service (CPS) for the food and beverage categories for January 2012 – December 2015. The Nielsen Company, 2016. The conclusions drawn from the Nielsen data are those of UNC and do not reflect the views of Nielsen. Nielsen is not responsible for and had no role in, and was not involved in, analyzing and preparing the results reported herein

*SSB* Sugar-sweetened beverage, *SD* Standard deviation

**Fig. 2:**
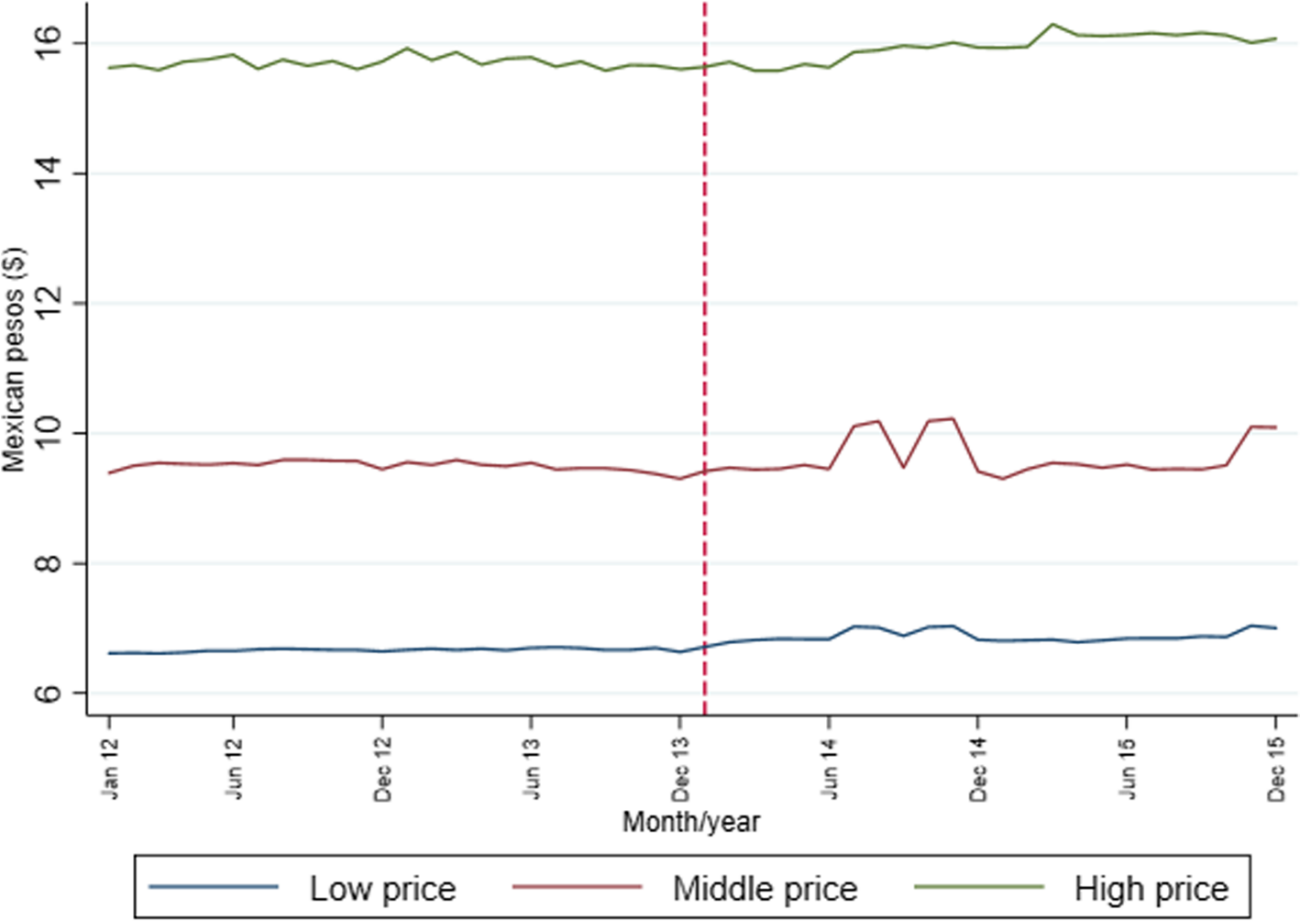
Average sugar-sweetened beverage prices per liter by price tertile. *Note:* Average prices are quantity weighted. Dotted red line for the tax implementation (January 2014). Source: Authors’ own analyses and calculations based on data from Nielsen through its Mexico Consumer Panel Service (CPS) for the food and beverage categories for January 2012 –December 2015. The Nielsen Company, 2016. The conclusions drawn from the Nielsen data are those of UNC and do not reflect the views of Nielsen. Nielsen is not responsible for and had no role in, and was not involved in, analyzing and preparing the results reported herein

Figure 2 presents the average price per liter by price tertile in urban Mexico from 2012 to 2015. This average price is around MP $7 for low-price SSBs, MP $10 for middle-price SSBs, and MP $16 for high-price SSBs. Prices across tertiles display no major change over time because we only calculated one set of prices for the full analytical period. However, the slight price increase across tertiles in 2014 and 2015 reflects that available products in these years tended to be more expensive than those in the pre-tax period (2012 and 2013).

**Fig. 3:**
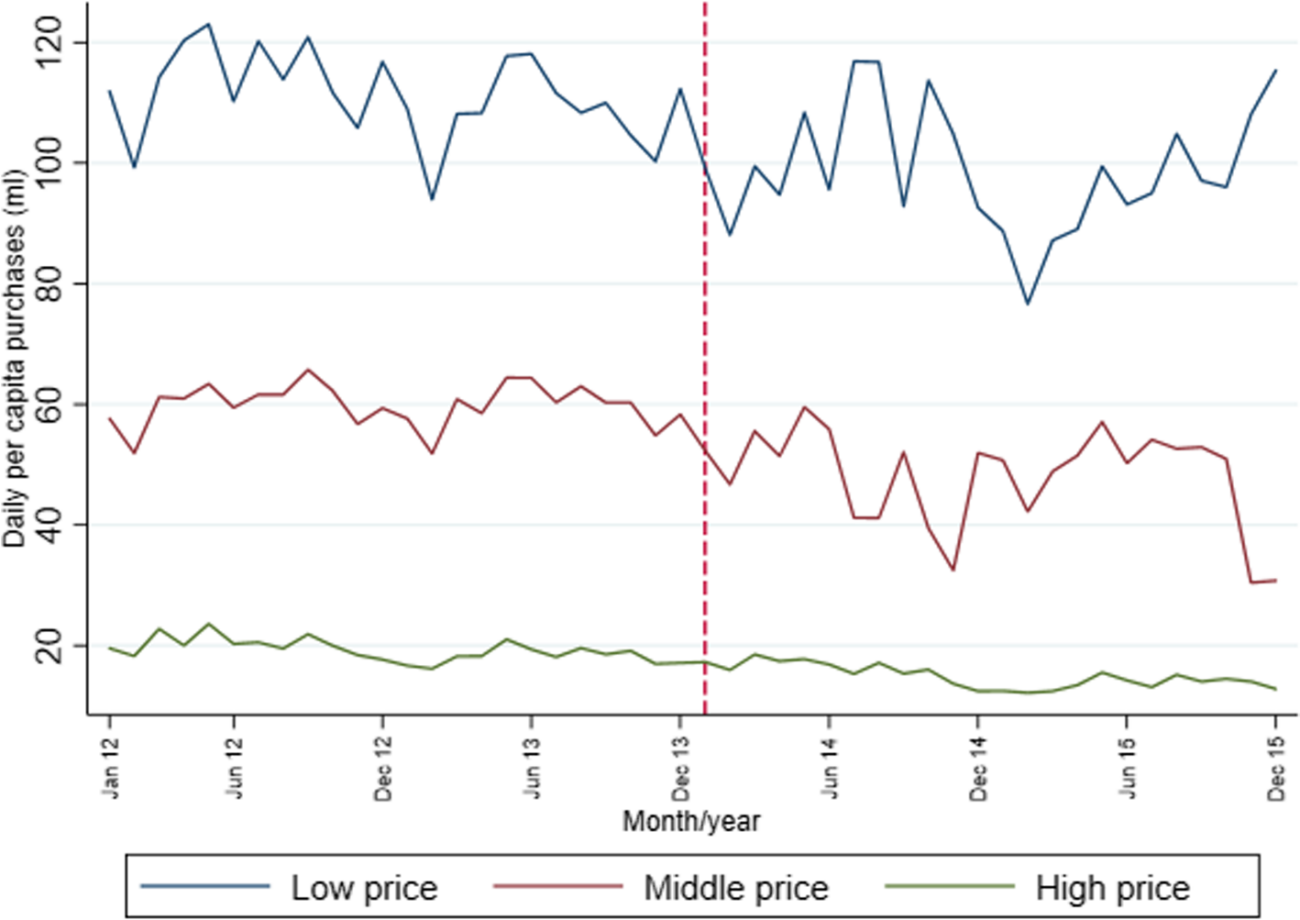
Sugar-sweetened beverage purchases by price tertile. *Note:* Population-weighted averages. Dotted red line for the tax implementation (January 2014). Source: Authors’ own analyses and calculations based on data from Nielsen through its Mexico Consumer Panel Service (CPS) for the food and beverage categories for January 2012 –December 2015. The Nielsen Company, 2016. The conclusions drawn from the Nielsen data are those of UNC and do not reflect the views of Nielsen. Nielsen is not responsible for and had no role in, and was not involved in, analyzing and preparing the results reported herein

Figure 3 presents the descriptive time trend of unadjusted monthly SSB purchases by SSB price tertile from 2012 to 2015. In line with Table 1, we see an inverse relationship between price tertiles and ml/capita/day SSB purchases. We observed more clear declines in SSB purchases after the tax implementation (i.e., January 2014) for middle-price SSBs. SSB purchases for high-price tertile display a sustained decline for the full period of 2012–15.

In Table 2, we present results of the linear regression models for each SSB price tertile. For each of these price tertiles, we report results for model 1 and model 2. For model 2, we additionally control for the percentage of products within the monthly price tertile of interest with a package size larger than one liter. For Model 1, after the SSB tax implementation, SSB purchases increased by 1.77 ml/capita/day and 0.11 ml/capita/day for the low and high price tertiles, respectively. In contrast, SSB purchases for the middle price tertile decreased by 13.79 ml/capita/day. However, only the latter result was statistically significant (*p* < 0.001), and thus SSB purchase changes under the low and high price tertiles were not different from zero. For model 2, SSB purchase changes for low and high price tertiles are similar compared to model 1 and remain statistically insignificant. Meanwhile in model 2, for the middle price tertile we see a statistically significant reduction of 10.8 ml/capita/day, slightly lower compared to model 1. For model 2, the coefficient for the percentage of products within the monthly price tertile with a package size larger than one liter was positive across all price tertiles but statistically significant (*p* < 0.001) only for the middle price tertile. Hence, a higher percentage of larger package sizes was associated with an increase in SSB purchases in the middle price tertile.

**Table 2:** Sugar-sweetened beverage purchase changes before and after the tax was implemented, by price tertile

**Tertiles of prices**	**Low price**				**Middle price**				**High price**			
	**Model 1**		**Model 2**		**Model 1**		**Model 2**		**Model 1**		**Model 2**	
	**Coefficient**	**SE**	**Coefficient**	**SE**	**Coefficient**	**SE**	**Coefficient**	**SE**	**Coefficient**	**SE**	**Coefficient**	**SE**
**Post-tax (2014–15)**	1.77	2.85	1.82	2.99	-13.79***	2.73	-10.80***	2.65	0.11	0.57	0.21	0.56
**Quarter 2**	10.08***	1.86	10.09***	1.88	5.45***	1.65	7.25***	1.66	2.10***	0.39	2.08***	0.39
**Quarter 3**	13.86***	2.11	13.90***	2.16	4.20*	2.07	5.60**	2.03	2.06***	0.39	2.17***	0.39
**Quarter 4**	13.58***	2.05	13.60***	2.11	-4.28*	2.01	-0.69	2.13	0.99**	0.36	1.08**	0.35
**Month(trend)**	-0.71**	0.23	-0.71**	0.23	0.06	0.19	-0.42	0.23	-0.17***	0.04	-0.16***	0.04
**Squared month (trend)**	0.00	0.00	0.00	0.00	-0.00	0.00	0.00	0.00	-0.00	0.00	-0.00	0.00
**Per capita monthly income**	0.02**	0.01	0.02**	0.01	-0.03***	0.01	-0.03***	0.01	-0.00	0.00	-0.00	0.00
**Unemployment rate**	0.04	1.37	0.05	1.37	-3.07*	1.29	-2.64*	1.27	-0.71**	0.23	-0.74**	0.23
**Monthly % products package > 1 L**			0.04	0.73			1.92***	0.41			0.14	0.09
**Number of observations**	2592				2592				2592			

Population-weighted estimates and robust standard errors in parentheses. Coefficients correspond to the marginal change in SSB purchases in ml/capita/day for the variable of interest. Models include city-fixed effects. *Source:* Authors’ own analyses and calculations based on data from Nielsen through its Mexico Consumer Panel Service (CPS) for the food and beverage categories for January 2012 – December 2015. The Nielsen Company, 2016. The conclusions drawn from the Nielsen data are those of UNC and do not reflect the views of Nielsen. Nielsen is not responsible for and had no role in, and was not involved in, analyzing and preparing the results reported herein

SE, standard error

^*^ *p* < 0.05

^**^ *p* < 0.01

^***^ *p* < 0.001

Additional figure A2 shows price trends from January 2012 to December 2015 based on the complementary pre- and post-tax price data. There was a consistent price increase before and after the tax implementation across all price tertiles; however, the largest average price increase equivalent to MP $0.97 per SSB liter corresponded to the low SSB price tertile. This price increase was MP $0.58 for the middle SSB price tertile and MP $0.87 for the high SSB price tertile.

## Discussion

We estimated changes in taxed beverage purchases by price tertile (low, middle, and high) after the SSB tax was implemented using linear regression models. Our results show an average reduction ranging between 10.8–13.79 ml/capita/day in purchases of taxed beverages from the middle-tertile of prices and non-significant changes in the low- and high-price tertiles.

Our results are in line with existing evidence of taxed beverage purchase reduction in urban Mexico. Our estimated purchase reduction between 10.8 and 13.79 ml/capita/day across taxed beverages from the middle-price tertile is similar to the overall average drop by 13.9 ml/capita/day in taxed beverage purchases in 2014 and 2015 in the study by Colchero et al. [4]. This consistency across studies suggests that the reduction in taxed beverage purchases in urban Mexico is entirely driven by changes in the middle-price tertile beverages.

A potential mechanism behind the purchase reductions only for middle-price SSBs might be that changes in SSB purchases in the tails of the distribution were unlikely. The null change in purchases of low-price SSBs may be explained by two reasons: 1) even though the largest price increases corresponded to low-price SSB (shown in additional Figure A2), these SSB remained the cheapest taxed beverages, and thus consumers might have no incentive to switch in the absence of cheaper SSB; 2) purchases in these low-price SSBs are already high as they account for about 59% of purchases, leaving less room for changes, i.e., consumers of more expensive SSB may not choose to switch to less expensive beverages based on their preferences. Meanwhile, the null purchase change across high-price SSBs might arise from their baseline low purchase levels as it accounts for only about 10% of all SSB purchases (< 20 ml/capita/day ml as already explained above), leaving little room for additional reductions. Based on these potential scenarios in the tails of the price distribution, consumers might have uniquely responded to the one-peso-per-liter SSB tax by reducing purchases of middle-price SSBs, as we found in this study. However, more research is needed to disentangle this potential mechanism.

Our results are in contrast to findings of the SSB tax evaluation in Barbados. While we only found purchase reductions in middle-price beverages and no changes across beverages in the remaining price tertiles, Alvarado et al. [9] for Barbados showed sales increases across middle-cost SSBs and sales reductions across low- and high-cost SSBs. It is worth noting that we should not expect comparable results for these two countries for factors such as different tax designs (i.e., per-unit tax in Mexico and ad valorem tax in Barbados) and potential differences in SSB market structures and SSB price elasticities.

Our study has some limitations. First, our findings are not generalizable to the country level as Nielsen CPS [10] is representative of Mexican cities with a population larger than 50,000 inhabitants, which accounted for 63% of the overall Mexican population and 75% of the overall food and beverage expenditure [4, 12]. Second, our analyses relied on a before-and-after approach, and thus we cannot claim we estimated the causal effect of the tax on purchases by price tertile. However, we included a set of controlling variables in our models to account for relevant SSB-demand drivers such as season and consumers’ income. Third, given the wide number of brands and package sizes, we did not analyze purchase changes in specific combinations of producers, brands, and package sizes. However, we controlled for the percentage of products with a package size larger than one liter within each monthly price tertile as a purchase driver. The observed SSB purchase reduction in this study could be associated with other policies implemented with the SSB tax. The SSB tax was approved along with an 8% ad-valorem tax on energy-dense non-essential packaged food. This tax could impact SSB purchases if there were complementarities between packaged food and SSB. Additionally, a marketing regulation was implemented in July 2014 to ban food and beverage ads on TV and movies targeted to children. Although there was adequate compliance, this policy is limited as children watch TV programs for adults. In light of these other policies and the lack of a control group, we cannot claim our findings represent causal estimates. Finally, we did not assess the substitution of SSB for untaxed beverages. Households might have responded to the tax implementation by increasing their consumption of cheaper (relative to SSB) untaxed beverages such as bottled plain water or tap water. Previous evidence showed substitution towards bottled plain water [3]. Lack of clean drinking water is unlikely to impact our results because only around 10% of urban households rely on tap water as the main drinking water source [12]. Moreover, in our analytical approach, we included fixed effects at the city level, which can account for clean water supply under the assumption that this supply did not change in the analyzed period.

The strengths of our study rely on the richness of the analytical data in Nielsen CPS [10]. These data include information on beverage purchases and prices that allowed us to model how households responded to the potential price changes after the SSB tax implementation in Mexico. Moreover, Nielsen CPS [10] provides information at the product level to identify both brands and their respective package size. This level of product disaggregation provided an essential source of price heterogeneity to build price tertiles. Our study represents the first analysis assessing how purchases of taxed beverages changed by beverage prices in Mexico.

From a public health perspective, findings from our study suggest that the current SSB tax has not been effective in reducing purchases across the full-price distribution. Thus, policymakers might consider adjustments to the SSB tax that might lead to purchase drops across all SSB. Public health experts in Mexico support doubling the amount of the current SSB tax to reinforce its expected health benefits [7]. Alternatively, policymakers can consider a redesign of the SSB tax towards sugar-density taxes where the tax burden depends on sugar, which is the nutrient of concern for SSBs, rather than volume as the current SSB tax in Mexico. In 2018, the United Kingdom and South Africa implemented some variation of sugar-density taxes. After the implementation of these taxes, the evidence in the United Kingdom and South Africa shows a potential product reformulation (i.e., sugar reductions) [18, 19] and drops in sugar from SSBs that exceeded the decrease in SSB purchases measured in terms of volume [19, 20].

## Conclusions

Our findings show only reductions across taxed beverages from the middle price tertile, which were consistent with the magnitude of the overall taxed beverage purchase reductions as in the study by Colchero et al. [4]. Based on these findings by Colchero et al. [4], Basto-Abreu et al. [22] predicted meaningful health-related improvements at the societal level linked to the SSB tax, which will lead to healthcare savings equivalent to USD $4 per each dollar spent on the tax implementation. Despite these positive outcomes associated with the SSB tax in Mexico, in this study, we did not find a reduction in purchases of taxed beverages from the low- and high-price tertile, which together concentrated the largest volume of purchases (≈70%) across taxed beverages in urban Mexico. Future studies should be conducted to test if the redesign of the current tax, by either doubling the tax amount or taxing sugar content, could reduce more effectively purchases across all SSB.

## Supplementary Information

**Additional file 1:** **Figure A1.** Number of months with available information at the brand-package size level.  

**Additional file 2:** **Figure A2.** Average sugar-sweetened beverage prices per liter by price tertile before and after the tax implementation.

**Additional file 3:** **Table A1.** Sugar-sweetened beverage purchases by household socioeconomic status and price tertile.

## Data Availability

The data that support the findings of this study are available from The Nielsen Company (Mexico) for its Mexico Consumer Panel Service (CPS) data (https://www.nielsen.com/us/en/clientlearning/consumer-panel-services/), but restrictions apply to the availability of these data, which were used under license for the current study, and so are not publicly available. We are unable to share the de-identified proprietary third-party dataset due to contractual data use agreements with the Nielsen Company. We did not have any special access privileges to the data (we paid for a data license) and got it through the data vendor, the Nielsen Company. For inquiries, please see: Consumer Panel Services – Nielsen (https://www.nielsen.com/us/en/client-learning/consumer-panel-services/).

## Abbreviations

ml: Milliliters
MP: Mexican pesos
Nielsen CPS: Nielsen Mexico Consumer Panel Service
SES: Socioeconomic status
SSB: Sugar-sweetened beverages
