# Supplementary material for: Changes in sugar-sweetened beverage purchases across the price distribution after the implementation of a tax in Mexico: a before-and-after analysis
Source: BMC Public Health. 2023 Feb 7;23:265. doi: 10.1186/s12889-023-15041-y (PMC9906831; doi:10.1186/s12889-023-15041-y)
Supplement: Supplementary file 1 — Additional file 1: Figure A1. Number of months with available information at the brand-package size level. [file 12889_2023_15041_MOESM1_ESM.docx]

**Figure A1. Number of months with available information at the brand-package size level**

**
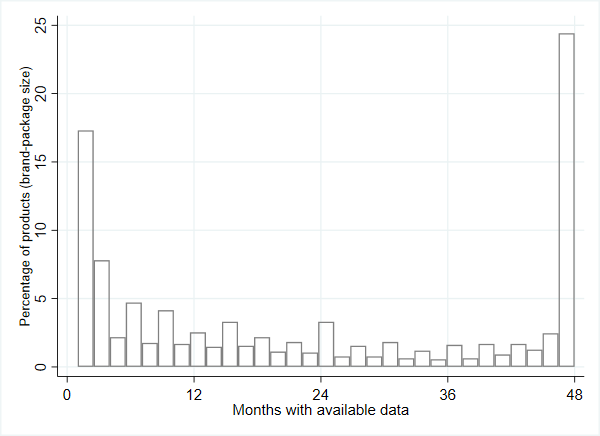
**

*Source*: Authors’ own analyses and calculations based on data from Nielsen through its Mexico Consumer Panel Service (CPS) for the food and beverage categories for January 2012 –December 2015. The Nielsen Company, 2016. The conclusions drawn from the Nielsen data are those of UNC and do not reflect the views of Nielsen. Nielsen is not responsible for and had no role in, and was not involved in, analyzing and preparing the results reported herein.
