# Supplementary material for: Changes in sugar-sweetened beverage purchases across the price distribution after the implementation of a tax in Mexico: a before-and-after analysis
Source: BMC Public Health. 2023 Feb 7;23:265. doi: 10.1186/s12889-023-15041-y (PMC9906831; doi:10.1186/s12889-023-15041-y)
Supplement: Supplementary file 2 — Additional file 2: Figure A2. Average sugar-sweetened beverage prices per liter by price tertile before and after the tax implementation. [file 12889_2023_15041_MOESM2_ESM.docx]

**Figure A2- Average sugar-sweetened beverage prices per liter by price tertile before and after the tax implementation**

**
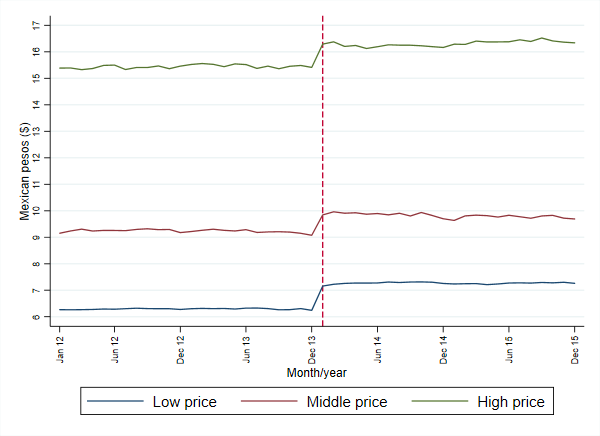
**

Note: Average prices are quantity weighted. Dotted red line for the tax implementation (January 2014). *Source:* Authors’ own analyses and calculations based on data from Nielsen through its Mexico Consumer Panel Service (CPS) for the food and beverage categories for January 2012 –December 2015. The Nielsen Company, 2016. The conclusions drawn from the Nielsen data are those of UNC and do not reflect the views of Nielsen. Nielsen is not responsible for and had no role in, and was not involved in, analyzing and preparing the results reported herein.
