# Supplementary material for: Changes in sugar-sweetened beverage purchases across the price distribution after the implementation of a tax in Mexico: a before-and-after analysis
Source: BMC Public Health. 2023 Feb 7;23:265. doi: 10.1186/s12889-023-15041-y (PMC9906831; doi:10.1186/s12889-023-15041-y)
Supplement: Supplementary file 3 — Additional file 3: Table A1. Sugar-sweetened beverage purchases by household socioeconomic status and price tertile. [file 12889_2023_15041_MOESM3_ESM.docx]

**Supplement.**

**Table A1. Sugar-sweetened beverage purchases by household socioeconomic status and price tertile**

|  |  | **2012-13**  **(pre-tax)** |  | **2014-15**  **(post-tax)** |  | **Difference 2014-15 vs 2013-2012** | |
| --- | --- | --- | --- | --- | --- | --- | --- |
| **SSB purchases (ml/per capita/day)** | **Socio-Economic Status (SES)** | **Mean** |  | **Mean** |  | **Mean** | **P-value** |
| **Low-SSB prices** | Low-SES households | 129.92 |  | 109.19 |  | -20.73 | 0.00 |
|  | Middle-SES households | 116.32 |  | 104.45 |  | -11.87 | 0.00 |
|  | High-SES households | 88.10 |  | 82.60 |  | -5.51 | 0.02 |
|  |  |  |  |  |  |  |  |
| **Middle-SSB prices** | Low-SES households | 64.12 |  | 46.46 |  | -17.65 | 0.00 |
|  | Middle-SES households | 60.75 |  | 50.80 |  | -9.95 | 0.00 |
|  | High-SES households | 58.51 |  | 44.85 |  | -13.66 | 0.00 |
|  |  |  |  |  |  |  |  |
| **High-SSB prices** | Low-SES households | 18.60 |  | 14.09 |  | -4.51 | 0.00 |
|  | Middle-SES households | 19.15 |  | 14.71 |  | -4.44 | 0.00 |
|  | High-SES households | 21.51 |  | 16.73 |  | -4.78 | 0.00 |
| **Observations** |  |  |  |  |  | 7776 |  |
| Note: SSB, sugar-sweetened beverages. Population weighted summary statistics. Mexican pesos. Source: Authors’ own analyses and calculations based on data from Nielsen through its Mexico Consumer Panel Service (CPS) for the food and beverage categories for January 2012 – December 2015. The Nielsen Company, 2016. The conclusions drawn from the Nielsen data are those of UNC and do not reflect the views of Nielsen. Nielsen is not responsible for and had no role in, and was not involved in, analyzing and preparing the results reported herein. | | | | | | | |
